# Do older people know why they take benzodiazepines? A national French cross‐sectional survey of long‐term consumers

**DOI:** 10.1002/gps.5307

**Published:** 2020-04-21

**Authors:** Edouard‐Jules Laforgue, Alexandra Jobert, Morgane Rousselet, Marie Grall‐Bronnec, FAN network, Pascale Jolliet, Fanny Feuillet, Caroline Victorri‐Vigneau

**Affiliations:** ^1^ Clinical Pharmacology Department Centre Hospitalier Universitaire de Nantes, University Hospital Nantes France; ^2^ Addictology and Liaison‐Psychiatry Department Centre Hospitalier Universitaire de Nantes, University Hospital Nantes France; ^3^ INSERM UMR 1246, SPHERE, Methods in Patients‐centered outcomes and Health Research Nantes and Tours University Nantes France; ^4^ Direction de la recherche Centre Hospitalier Universitaire de Nantes, University Hospital Nantes France; ^5^ French Addictovigilance Network France

**Keywords:** aged, benzodiazepines, dependence, treatment knowledge, Z‐drugs

## Abstract

**Objectives:**

Benzodiazepines and non‐benzodiazepine hypnotics (or Z‐drugs) (BZD/Z) are widely prescribed for older patients despite major side effects and risks when chronically used. The patient's understanding of the treatment is one of the keys to good adherence. The purpose of the study was to assess the knowledge of BZD/Z treatment among older people who were taking BZD/Z for the long term by studying the concordance between the declared reason for taking BZD/Z and its indication.

**Methods:**

This was a cross‐sectional, pharmacoepidemiologic ancillary of a national study. Data were collected through a semi‐structured interview. All patients from the main study were included. “Good knowledge” was considered when patients gave an indication for each BZD/Z that was similar to its marketing authorisation. Univariate and multivariate analyses were carried out to adequately determine profiles and characterise associations.

**Results:**

More than half of the patients (61.6%) had a good knowledge regarding their treatment. The presence of a psychiatric disorder, a mean duration of BZD/Z use of less than 120 months, a desire to stop treatment, educational status and number and type of BZD/Z used were significantly associated (*P* < .05) with good knowledge. In the multivariate analysis, only a psychiatric disorder, educational status and taking at least one hypnotic drug were associated with good knowledge.

**Conclusions:**

At the time of shared medical decision, it appears essential to improve the knowledge of the treatment by the patient. The rate of patients with good knowledge of their BZD/Z treatment remains low and even lower than what was previously found in the literature for other drug classes. In contrast to patients with good knowledge, these data highlight the characteristics of patients with poor knowledge of their BZD/Z treatment, which may allow populations at risk to be targeted and enable education measures to be strengthened.

**Key points**

- Of the 1023 patients, the majority 61.6% have a good knowledge of the treatment's indication—a rate that remains low.
- Declaring a psychiatric disorder, educational status and to take at least one Z‐drug were associated with a good knowledge of treatment.
- Treatment such as hypnotics is more likely associated with a good knowledge than anxiolytic BZD.
- Only 11% of patients report a current psychiatric trouble, while all patients have been using benzodiazepines for more than 3 months

## 1 INTRODUCTION

Benzodiazepines (BZD) and related drugs (Z‐drugs) are widely prescribed,^1^ especially in France, where they are among the most prescribed drugs, with more than 2 millions of long terms users among people aged of 65 years old or more in 2007.^2^ This high prevalence—approximately a fifth of the French aged population—is astonishing given (a) the restricted indications: delirium tremens prevention and symptomatic treatment of severe and/or invalidating anxiety or transient or occasional insomnia and (b) the time limited: 4 weeks for hypnotics and 12 weeks for anxiolytics.

Despite their initial good efficacy for anxiety and insomnia, BZD/Z use carries major concerns about side effects such as anterograde amnesia, altered psychomotor functions, behavior, memory trouble, altered conscience state and dependence^3^ when chronically used. These risks are increased by altered pharmacokinetic and pharmacodynamic parameters in older people.^4^

A previous survey in 1996 shows that **previous failure to stop taking the medication could explain 32% of continuous prescriptions of hypnotics.** ^5^ Given the increase in prescriptions and the difficulty of discontinuing these medications, the French health authority in 2010 established recommendations to help practitioners stop BZD/Z use.^6^ Beyond progressively decreasing doses and stopping, the recommendations emphasise that the presence of a psychiatric disorder, comorbid dependence and previous unsuccessful attempts to stop are factors that could lead to unsuccessful cessation of treatment.

In a 2014 study, Gérardin et al^3^ found a rate of 44% of unsuccessful attempts to stop treatment in older patients. **Among the factors that could explain why older people failed to stop, dependence in older people** ^7^ **and lack of knowledge about the treatment** seemed to be major concerns.

Knowing the indication of prescribed drugs is essential and contributes to providing patients with a central position regarding their health care. With this information, patients can address their difficulties to the practitioner to choose the best therapeutic option. The concept of “shared decision making”^8^ tends to be increasingly important. Treatment knowledge has been investigated in several studies with heterogeneous methodologies and results.^9^, ^10^, ^11^, ^12^

The literature does not include any study that directly examines specific knowledge of the indications of BZD/Z treatment among older people. The aim of this exploratory study was to assess the knowledge of the indications of BZD/Z in people aged 65 years or older who had been taking BZD/Z. Thus, we estimate the prevalence of patients with good knowledge of the indications of BZD/Z treatment(s).

## 2 MATERIALS AND METHODS

### 2.1 Study oversight

This study is an explanatory ancillary study of a national observational prospective study conducted by the French Addictovigilance Network (FAN) from March 2012 to December 2015. The FAN is the French official network of 13 drug dependence evaluation and information centres (CEIP‐A) throughout France, in charge of drug monitoring under the responsibility of the French National Health Product Agency (ANSM). The main study aimed to assess and characterize BZD/Z dependence in older people (results are being submitted for publication) and was funded by a grant from the French Ministry of Health (PHRC 2010). It was monitored by a pluridisciplinary steering committee composed of pharmacologists (one from each CEIP‐A), the Narcotics and Psychotropic National Committee chairman, psychiatrists who specialise in addiction, biostatisticians, pharmacists, general practitioners (GP) and geriatricians.

This study was approved by the local health ethics committee, the CCTIRS (Comité Consultatif sur le Traitement de l'Information en matière de Recherche dans le domaine de la Santé) and the CNIL (Commission Nationale de l'Informatique et des Libertés). All participants provided written informed consent in accordance with the Declaration of Helsinki. The study is registered as NCT01920581.

### 2.2 Patients

To be eligible, patients had to be 65 or older, be treated with benzodiazepines or Z‐drugs in ambulatory care for at least 3 months (the maximal duration of an anxiolytic prescription in France) and give informed consent. Patients who were not fluent in French and/or with major cognitive impairments that prevented them from understanding the questions were excluded from the study.

### 2.3 Study procedures

The patient's recruitment occurred where patients received their treatment in pharmacies, which necessarily included eligible patients. All patients were then interviewed by phone by a trained interviewer without knowledge of the practitioner and without disturbing the patient‐doctor relationship.

The data included sociodemographic data, health problems (physical health problems and psychiatric issues), tobacco and alcohol consumption, treatments and prescribers, BZD/Z data (number of treatments, ATC classification, indication declared, maximum duration of treatment, prescriber) and dependence perception items towards BZD/Z treatment (limitations by prescriber, bypassing prescriptions, socio‐affective negative consequences, desire to stop). The data were self‐reported by the patient. In order to be as close as possible to the patient's real‐life, minimal help was provided if the subject requested clarifications (eg, when asked “do you have current psychiatric problems?” the interviewer specified “as depression or anxiety”). The number of non‐BZD/Z drug treatments was also requested.

BZD/Z treatments were divided into several groups according to their mentioned indications in the summary of product characteristics and the ATC classification (a) anxiolytic treatments (N05B): benzodiazepines such as alprazolam, diazepam, (b) hypnotic treatments (N05C): Z‐drugs and lormetazepam and (c) other treatments: clonazepam (N03A), tetrazepam (M03).

The knowledge of the treatment was evaluated by a direct question from the evaluator to the patient, mentioning the BZD/Z treatment concerned: “Can you tell me why you are taking this treatment?” The expected answers were: for anxiety, stress, for N05B category; to sleep for N05C category; seizure for N03A category and myorelaxant for M03 category—according to the drugs approvals. Patients were rated as having (a) good knowledge in cases of good agreement between their described reason for taking the treatment and the treatment indications and (b) as having bad knowledge in cases of bad agreement. In cases in which patients were taking multiple BZD/Z medications, they were considered to have good knowledge if there was no bad agreement for any of the medications.

### 2.4 Outcomes

The main objective was to estimate the prevalence of patients with good knowledge of the indications of BZD/Z treatment(s).

The secondary objective was to characterize patient profiles associated with good knowledge of the indications of BZD/Z treatment(s).

### 2.5 Statistical analyses

Descriptive statistical analyses of the sociodemographic and clinical characteristics were conducted for the entire sample. Continuous variables are described as the mean and SDs, and categorical variables are presented by numbers and percentages.

Univariate analyses were conducted to explore the associations between the patient's good and bad knowledge status at inclusion and the set of variables mentioned above. We used Student's test for quantitative variables and *χ* ^2^ (or if not applicable Fischer's exact test) for categorical variables. The threshold of significance was fixed at *P* < .05.

Multivariate analyses were performed using an iterative selection procedure to select the variables that were significantly associated with the good knowledge status, as assessed by the likelihood ratio test (candidate for the model were variables associated with good or bad knowledge in univariate analyses with a *P* < .20 criterion in the univariate analysis‐variables for the individuals BZD/Z were excluded). The discrimination of the final logistic model was assessed using the area under the receiver operating characteristic curve, and the model's goodness‐of‐fit was assessed using the Hosmer‐Lemeshow test.

The calculations and statistical tests were carried out with the software R (version 3.4.3).

## 3 RESULTS

In total, 1023 patients recruited by more than 250 pharmacies in France were included in this study (Table 1).

**TABLE 1:** Sociodemographic characteristics and univariate analysis of BZD/Z knowledge

	Total (n = 1023)	Bad knowledge (n = 393)	Good knowledge (n = 630)	*P*‐value
Sex (male)	272/1023 (26.5%)	110/393 (28.0%)	162/630 (25.7%)	.42
Mean age (SD)	75.5 (±6.7)	75.92 (±6.6)	75.20(±6.7)	.09
Educational level:				.009**
Post bachelor's degree education	115/982 (11.7%)	30/370 (8.1%)	85/612 (13.9%)	
Bachelor's degree	130/982 (13.2%)	44/370 (11.9%)	86/612 (14.1%)	
Technical diploma	116/982 (11.8%)	40/370 (10.8%)	76/612 (12.4%)	
No diploma or a middle school diploma	621/982 (63.2%)	257/370 (69.4%)	364/612 (59.4%)	
Living alone (yes)	436/1021 (42.7%)	163/393 (41.5%)	273/628 (43.5%)	.53
Current health issues (yes)	579/1017 (56.9%)	214/392 (54.6%)	365/625 (58.4%)	.23
Current psychiatric issues (yes)	117/1015 (11.5%)	34/391 (8.7%)	83/624 (13.3%)	.02*
Ever smoker (yes)	297/1020 (29.1%)	109/393 (27.7%)	188/627 (30.0%)	.43
Probable alcohol dependence (yes)	195/1021 (19.1%)	78/392 (19.9%)	117/629 (18.6%)	.65
Mean number of treatments (n *≥* 4) other than BZD/Z drugs	503/1023 (49.2%)	196/393 (49.9%)	307/630 (48.7%)	.72
Self‐prescription or self‐medication (yes) for BZD/Z drugs	40/1015 (3.9%)	17/393 (4.3%)	23/622 (3.7%)	.58
Mean duration of BZD/Z treatment (>120 months)	159.7 (± 127.3)	176/393 (45.5%)	238/630 (38.1%)	.02*
BZD prescriber (only GP)	959/1017 (94.2%)	375/393 (95.5%)	584/624 (93.6%)	.22
BZD/Z number (≥2)	187/1023 (18.3%)	25/393 (6.4%)	162/630 (25.7%)	<.001***
Only N05B prescriptions	524/1023 (51.2%)	351/393 (89.3%)	173/630 (27.5%)	<.001***
Only N05C prescriptions	323/1023 (31.6%)	14/393 (3.6%)	309/630 (49.1%)	<.001***
N05B + N05C combined	149/1023 (14,6%)	3/393 (0.8%)	146/630 (23.2%)	<.001***
Zolpidem	251/1023 (24.5%)	5/393 (1.3%)	246/630 (39.0%)	<.001***
Zopiclone	147/1023 (14.4%)	4/393 (1.0%)	143/630 (22.7%)	<.001***
Alprazolam	157/1023 (15.3%)	70/393 (17.8%)	87/630 (13.8%)	.08
Bromazepam	216/1023 (21.1%)	110/393 (28.0%)	106/630 (16.8%)	<.001***
Lorazepam	209/1023 (20.4%)	128/393 (32.6%)	81/630 (12.9%)	<.001***
Oxazepam	72/1023 (7.0%)	50/393 (12.7%)	22/630 (3.5%)	<.001***

Dependence perception items	n = 976	n = 375	n = 601	
Limitation of prescription by prescriber (yes)	129 (13.2%)	40 (10.7%)	89 (14.8%)	.06
Bypassing the prescription (yes)	265 (27.2%)	92 (24.5%)	173 (28.8%)	.14
Entourage issues (yes)	71 (7.3%)	31 (8.3%)	40 (6.7%)	.34
Desire to stop treatment (yes)	672 (68.9%)	241 (64.3%)	431 (71.8%)	.01*

* *P* < .05.

** *P* < .01.

*** *P* < .001.

The participants' ages ranged from 65 to 95 years, and three‐quarters were women. One‐quarter of this population had a baccalaureate degree or higher, and most were retired. Forty percent lived alone, and they were divided between urban (45%) and rural populations (55%). Almost all of the patients were autonomous, especially for the management of their treatment.

Half of the patients declared a health problem and approximately 10% reported a psychiatric problem. Regarding alcohol consumption, 80% had a low risk or no risk of dependence. Almost 30% were currently smokers. Detailed results concerning dependence will be published with results of the main study. Almost all the subjects received treatments other than BZD/Z.

The 1023 patients took 1221 BZD/Z treatments (average: 1.2 per patient). The most frequently prescribed treatments are described in Table 1.

Bypassing a prescription, that is, using an alternative means to obtain the drug, was reported by 1 patient in 3, and the vast majority of patients had desire to stop treatment.

Of the 1023 patients, 61.6% (n = 630) had good knowledge of their treatment's indication, while 38.4% (n = 393) had poor knowledge.

In the population with good knowledge, patients had significantly more reported psychiatric problems (*P* = .02), a maximum treatment duration more likely shorter than 120 months (*P* = .02), more frequent desire to stop BZD/Z (*P* = .01) and fewer than two treatments per BZD/Z (*P* < .001). Patients with good knowledge used significantly solely (*P* < .001) or combined hypnotic BZD (*P* < .001). The educational level differed significantly between the two groups (*P* < .01). Dependence items were more prominent in the group with good knowledge, but the difference was not significant (Table 1).

Table 2 shows the data for the multivariate analysis of the BZD/Z fit. In our model, three variables increased the probability of a good match between the treatment and the declared indication: declaring a psychiatric issue (*P* = .02), having a post bachelor's degree education (*P* < .001), a bachelor's degree or a technical diploma (*P* < .01) and taking only hypnotics (*P* < .001) or combined with anxiolytics (*P* < .001).

**TABLE 2:** Multivariate analysis of good knowledge of treatment (N = 970)

	Adjusted OR	95% CI (OR)	*P*‐value
Current psychiatric issues (yes vs no)	1.99	[1.14; 3.49]	.02*
Educational level			
Post bachelor's degree education	2.92	[1.69; 5.04]	< .001***
Bachelor's degree	1.19	[0.68; 2.07]	.55
Technical diploma	2.08	[1.23; 3.52]	< .01**
No diploma or a middle school diploma	Ref	‐	‐
Anxiolytics/hypnotics use			
Only anxiolytics	Ref	‐	‐
Only hypnotics	52.18	[29.32; 92.86]	< .001***
Anxiolytics and hypnotics	105.74	[33.02; 338.65]	< .001***

*Note*: Hosmer and Lemeshow's test of this model showed a *P =* 0.65, reflecting the match between reality and the model's predictions. The AUC was 0.88, which indicates that the model had good power of discrimination.

Abbreviations: 95% CI, 95% confidence interval; OR, odds ratio; Ref, class of reference.

* *P* < .05.

** *P* < .01.

*** *P* < .001.

## 4 DISCUSSION

One of the main contributions of this study is its description of the prevalence of good and bad knowledge about BZD/Z indications among older patients. This prevalence specifically in relation BZD/Z had never been clearly described before. The inability to report long‐term treatment use to a health professional can be extremely deleterious or even dangerous.^13^ Our study found that less than two‐third of older patients were able to correctly report their reasons for taking BZD/Z, a rate that remains low.

A Chinese study^11^ of 412 patients aged 60 years or older with at least one chronic disease who were recruited from general outpatients clinic found 76.2% understood the indications for their treatments when assessed after a regular medical consultation. Patients were asked “why are you taking this medication?” on the *Medicine Knowledge Assessment Form*. However, the drug classes and type of chronic disease were not detailed. A study of a population of community‐dwelling people aged 65 years or older in the United States was conducte^14^ to assess through a structured interview, patients' knowledge of the purpose of their treatment. Patients were recruited as part of a therapeutic education programme and came to the centre for assessment at the same time as they were receiving the programme. Of the 375 patients, 87% were able to cite the indication their medications. Here too, we have no information of the class of drug evaluated or the type of disease. Similar rates were observed in the study by Jaye et al,^10^ where patients were recruited from a general practice. A questionnaire was given and fulfilled by the patient prior to the appointment with a doctor or nurse. The population consisted of 344 patients older than 16 years old, 90% of whom were on long‐term treatment (more than 3 months). Treatment knowledge was assessed using the question “why did the doctor prescribe?” and knowledge was considered good if there was agreement between the patient's answer and the purpose stated in the medical record. The result was that 87% of subjects knew the indications of all their treatments but without any information on the drug classes evaluated or the diseases presented by the patients. Over a period of 2 months, Chung found in another American study of 77 patients over 65 years of age seen in the emergency department, that 83.3% of the drug indications were correctly identified according to the *Physician*' *Desk Reference*s.^15^ Akici's study^12^ of more than 1600 postoperative patients recruited from a primary healthcare department showed much lower results. In a face‐to‐face questionnaire, patients were asked about the names and main effects of drugs. In the group of patients who could not recall the name of drugs (almost 90% of the sample), only 46.5% could recall main effects of the treatment. CNS drugs represent 3.2% of all drugs in the sample. The heterogeneous methodology used in previous studies may account for the difference in rates concerning treatment knowledge. Generally, patients were recruited at their GP's office or the day after their hospital discharge, whereas in our study, patients were contacted at home a few days later days after the recruitment by the pharmacist. The time elapsed between recruitment and the telephone interview may explain the poorer results than in the literature, with the exception of Akici's study. On the other hand, this gives our study a quality that is closer to the patient's “daily life” and is free from any evaluation bias (eg, the patient is better able to recall treatment name and indication just before or just after a medical appointment). The methodology to investigate and assess good knowledge of the treatment is heterogeneous: main effect, supposed reason for prescribing, etc…and criteria of judgement are poorly described except the in the Jaye's and Chung studies (agreement with medical record and *Physician*' *Desk References, respectively*), our study is the only one to assess agreement based on marketed indication. Although the off‐label is not evaluated, it is indicative of what patients know about their treatment regardless of their doctor‐patient relationship. Finally, drug classes and chronic diseases are not generally described in studies results. Our study is the only one to focus on benzodiazepines, drugs which have cognitive side effects that can alter responses.

As confirmed by the literature,^11^ educational level is an explanatory factor correlated with treatment knowledge. This factor can be linked with the patient's level of knowledge about disease. Surprisingly, in contrast to the existing literature,^10^, ^11^, ^16^ age was not associated with worse knowledge of drug indications.

The time gap between the doctor filling the prescription and the collection of data may have affected the lower knowledge rates. In contrast, at recruitment time, the patients were informed that they would be contacted specifically for a study on their BZD/Z medications and were asked to have their prescriptions with them at the call time, which suggests that the rates found may be overestimated.

Treatments such as hypnotics were more likely to be associated with good knowledge than anxiolytic. Not only did almost all the patients who took only hypnotic drugs have good knowledge, but the rates were even higher among patients who took both anxiolytics and hypnotics. These results are interesting because generally, the higher the number of treatments prescribed, the weaker the patient's knowledge about each treatment.^14^, ^15^, ^17^, ^18^ However, the multivariate analysis warrants a cautious interpretation of the odds ratios due to the small number of observations in some cases.

Insomnia is widespread (its prevalence in general practice is between 46% and 61%^19^, ^20^, ^21^, ^22^) and has concrete symptoms that can have an immediate and major repercussion on quality of life. The patients' excellent knowledge of hypnotics can be explained by the different measures implemented by health policies, including warnings about short‐term action and the resulting need for administration just before bedtime that help patients integrates the motif “for sleeping” with this prescription. In addition, anxiolytics may have several indications while hypnotics have only one. This factor may explain the poorer knowledge of anxiolytic treatment. Meanwhile, anxiolytic prescription use was also related to limitations of prescription duration and instructions for prescription plans.

It is very surprising that only 11% of the patients reported a current psychiatric illness, although all of them had been using benzodiazepines for more than 3 months, which is already longer than the recommended prescription duration. Psychiatric disorders can be difficult for the patients themselves to recognize. This lack of insight may be explained by the pathology itself, by cognitive alterations in older people or by cultural bias.^23^ One possible source of bias underlying underestimation is the choice to focus on current psychiatric issues, thereby failing to report essential underlying factors. For example, anxiety can be a symptom of psychiatric disorders as major depressive disorder, generalised anxiety disorder, etc.; meanwhile, BZD treat symptoms and not aetiologies.^6^ It is possible that because the patient does not recognise and mention a psychiatric disease, only symptomatic treatment is prescribed, and information the more general underlying disorder is not noticed or integrated. In such cases, the result can be a very long mean duration of BZD/Z prescription (durations up to 10 years were observed in this study!), suggesting that prescription is continually renewed as the years go by, and BZD/Z are integrated as a “routine” of daily life without an understanding of the initial condition. This view is confirmed by the fact that mean prescription durations >120 months were more likely to be associated with poor knowledge. On the other hand, when a patient is able to recognise and self‐report a psychiatric issue, it becomes clear that he is more likely to know why he or she is taking the medication.

Moreover, the desire to stop treatment was significantly more common in the good knowledge group. We can assume the existence of a kind of paradox in which patients do not know why they are taking the drug but do not want to stop it, and ultimately, that lack of knowledge makes discontinuing treatment more difficult.

The database of 1023 patients included in our multicentre study is particularly interesting and allowed a valid statistical analysis. Furthermore, it partly answers a crucial question that is known as a main factor in the successful cessation of treatment: do older people know why they take BZD/Z?

Comparing our older BZD/Z consumers to the general population, we found similar characteristics in terms of the sex ratio, educational level, prescribing practitioner and prescribed drugs, indicating that our sample was representative.^24^

Our methodology was original compared to other studies. The fact that patients were recruited not at hospital discharge or from a GP's office but at their pharmacies and were contacted at their homes a few days later imparts a quality that is closer to the patient's “daily life” and is freed from evaluation bias.

Our study has several limitations. First, the definition of treatment knowledge is non‐unambiguous as seen with heterogeneous methodology in the previous literature. We deliberately have chosen an agreement between self‐declared indication and marketed approvals, aware of the limitation of such a soft judgement criterion for an exploratory study. In addition, non‐anxiety or non‐hypnotic uses of BZD/Z were not evaluated (due to very marginal data in the population and our sample [less than 10 patients]), nor was off‐label use evaluated. Nevertheless, we did not explore specifically in this part of the study knowledge about side‐effects, dosage or even the mode of administration, … in order to not making the interview too intrusive and not interfering in the doctor‐patient relationship. Despite the recruitment taking place in the whole of France, the health policies and specific care organizations do not make it possible to generalise the results to the other countries. The patients were recruited on the basis of BZD/Z use and were informed of the study topic. This factor may have influenced the patients' answers. Although we focused on current psychiatric issues, it is possible that the rates of psychiatric issues declared would have been higher if the patients had been asked about past periods. Patients' cognitive functions were not assessed prior to inclusion in the study. However unlikely, biased responses from patients with mild to moderate cognitive impairment cannot be excluded.

## 5 CONCLUSION

Prescriptions of BZD/Z in older patients represent a major current challenge for doctors. This particularity is well illustrated in the difficulty of stopping BZD/Z use. In addition to dependence on the treatment, another possible reason for unsuccessful attempts to stop treatment is the patients' lack of knowledge about these medications. These data highlight the characteristics of patients with poor knowledge of the drug, which may allow populations at risk to be targeted and enable education measures to be strengthened. This is particularly applicable for patients who take a treatment despite declaring no current health problems; although the treatment can achieve its objectives (anxiolysis and sedation), the aetiological problem does not disappear despite the improvement of physical, functional or paraclinical signs. These measures ultimately aim to improve adherence through improved drug knowledge.

## CONFLICT OF INTEREST

The authors declare no conflicts of interest.

## DATA AVAILABILITY STATEMENT

Given the confidentiality of the data, our ethics committee (GNEDS) is preventing us from making our data set publicly available. However, we are willing to make our data available upon request as we consider that it is important for open and reproducible science, and thus we will ensure that all interested and qualified researchers will be able to be granted access. Furthermore, Anne Omnès (anne.omnes@chu-nantes.fr) is the contact for data requests, and she will able to approve and distribute our data upon request.
